# Efficacy of the EZ-IO^® ^needle driver for out-of-hospital intraosseous access - a preliminary, observational, multicenter study

**DOI:** 10.1186/1757-7241-19-65

**Published:** 2011-10-26

**Authors:** Richard Schalk, Uwe Schweigkofler, Gösta Lotz, Kai Zacharowski, Leo Latasch, Christian Byhahn

**Affiliations:** 1 Clinic of Anesthesiology, Intensive Care Medicine and Pain Therapy, J.W. Goethe-University Hospital Frankfurt, Germany; 2 Department of Trauma and Orthopedic Surgery, BG Unfallklinik, Frankfurt, Germany; 3 Department of Public Health, Municipality of Frankfurt, Germany

**Keywords:** Intraosseous access, EZ-IO^® ^needle driver, Emergency medicine

## Abstract

**Background:**

Intraosseous (IO) access represents a reliable alternative to intravenous vascular access and is explicitly recommended in the current guidelines of the European Resuscitation Council when intravenous access is difficult or impossible. We therefore aimed to study the efficacy of the intraosseous needle driver EZ-IO^® ^in the prehospital setting.

**Methods:**

During a 24-month period, all cases of prehospital IO access using the EZ-IO^® ^needle driver within three operational areas of emergency medical services were prospectively recorded by a standardized questionnaire that needed to be filled out by the rescuer immediately after the mission and sent to the primary investigator. We determined the rate of successful insertion of the IO needle, the time required, immediate procedure-related complications, the level of previous experience with IO access, and operator's subjective satisfaction with the device.

**Results:**

77 IO needle insertions were performed in 69 adults and five infants and children by emergency physicians (n = 72 applications) and paramedics (n = 5 applications). Needle placement was successful at the first attempt in all but 2 adults (one patient with unrecognized total knee arthroplasty, one case of needle obstruction after placement). The majority of users (92%) were relative novices with less than five previous IO needle placements. Of 22 responsive patients, 18 reported pain upon fluid administration via the needle. The rescuers' subjective rating regarding handling of the device and ease of needle insertion, as described by means of an analogue scale (0 = entirely unsatisfied, 10 = most satisfied), provided a median score of 10 (range 1-10).

**Conclusions:**

The EZ-IO^® ^needle driver was an efficient alternative to establish immediate out-of-hospital vascular access. However, significant pain upon intramedullary infusion was observed in the majority of responsive patients.

## Background

Establishing immediate vascular access is a crucial step in the treatment of critically ill patients. Therefore, patients with difficult venous access remain a challenge for paramedics and emergency physicians. Using standard peripheral intravenous (IV) catheters often requires multiple attempts, is time consuming and may be ultimately unsuccessful. Unfavorable co-factors, such as hypovolemia, difficult access to the patient or poor lighting, can further aggravate these difficulties.

Intraosseous (IO) access represents a reliable alternative and is increasingly being used in the prehospital setting [1-5] and in the emergency department [6,7]. Further, it is an explicitly recommended procedure in the current guidelines of the European Resuscitation Council when intravenous access "cannot be established within the first 2 min of resuscitation" or is otherwise "difficult or impossible" [8]. Using manual screw needles, the Bone Injection Gun (BIG) or a semi-automatic insertion device (EZ-IO^®^), the procedure has been demonstrated to be quick, safe and efficient [1-7,9-12]. However, data regarding out-of-hospital IO access established by relative novices is limited.

The aim of the study was to prospectively evaluate the efficacy of a battery-powered needle driver (EZ-IO^® ^Intraosseous Infusion System, Vidacare Inc. Shavano Park, TX, USA) used by novice users - paramedics and emergency physicians - in patients with difficult vascular access in the prehospital environment of three emergency medical services in Germany and Switzerland.

## Materials and methods

### Study design and setting

All prehospital needle insertions with the EZ-IO^® ^device performed by paramedics and emergency physicians within a 24 month-period were recorded by means of a standardized questionnaire. The investigation was exempted by the J.W. Goethe University's ethics committee from formal review. The emergency medical services (EMS) that participated in the study consisted of six mobile intensive care units, each manned with one paramedic and one board-certified emergency physician that operated 24/7, and one rescue helicopter (one paramedic, one emergency physician, operated during daylight hours only) in the districts of Frankfurt/Main and Bad Kreuznach in Germany, and a paramedic-based ambulance system (four units manned with two paramedics each, operated 24/7) in the Swiss canton Appenzell-Innerrhoden. The universal access numbers in Germany and Switzerland are 112 and 144, respectively.

IO access was considered at the sole discretion of the paramedic or emergency physician in charge. In particular, there was no study protocol directive to first undertake a certain number of venous access attempts before choosing the IO route.

Since 2008, all paramedics and emergency physicians providing services in these areas undergo training in IO access with the EZ-IO^® ^that consists of a 15 min hands-on manikin training on a yearly basis.

### EZ-IO^® ^Intraosseous Infusion System

The EZ-IO device is a sealed medical drill to establish IO access. The lithium battery is capable of performing a minimum of 500 needle insertions. The needles come in one diameter (15 Gauge), but different lengths of 15 mm (pediatrics, 3-39 kg), 25 mm (adults, > 40 kg), and 45 mm ("excessive tissue"). When touching the bone at least 5 mm of the needle must be visible to ensure that the tip of the needle sufficiently enters the medullary space. Further applying gentle, steady downward pressure, a "pop" is felt upon entry of the needle into the medullary space.

### Data collection and processing

Standardized questionnaires were distributed to all EMS operators that participated in the study and returned by mail to the principal investigator immediately after the rescue mission. In case of further inquiries the rescuer was contacted over the phone. The questionnaires were anonymized in terms of patient's personal data, and location of the mission. Recorded data recorded included demographics, indication for IO needle use, access site, number of previously placed IO needles, pain upon fluid or drug administration via the needle, and subjective satisfaction regarding handling of the device and ease of needle placement.

### Outcome measures

The primary outcome variables were overall placement success of the IO needle. Needle placement was considered successful when passive bone marrow reflux was observed or bone marrow could be aspirated. Secondary variables comprised the site of IO access, number of previous attempts to establish intravenous access if applicable, immediate procedure-related complications, the level of previous experience with IO access, and operator's subjective satisfaction with the device.

### Statistics

After testing for Gaussian distribution, all data are expressed as median and range, or number and percent as appropriate. All statistical calculations were performed with IBM-based software packages (Microsoft Excel 2007, Microsoft Deutschland GmbH, Unterschleißheim, Germany, and GraphPad InStat Version 3.06; GraphPad Software Inc., San Diego, CA, USA).

## Results

**Figure 1:**
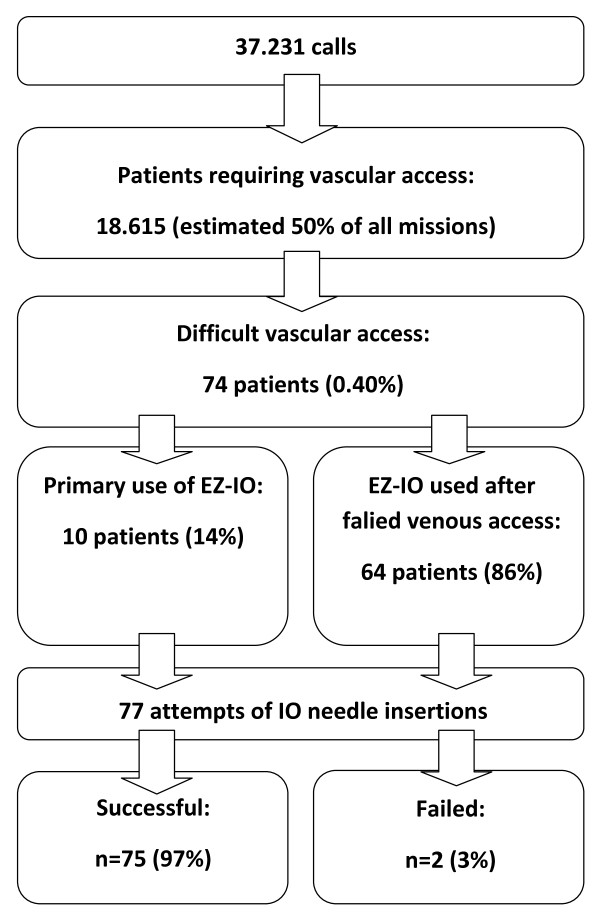
**Study flow chart**.

During a 24 month-period, the participating EMS units responded to 37,231 calls. The EZ-IO^® ^was used for 77 needle insertions (access site proximal tibia: n = 75; distal tibia: n = 2) in 69 adult patients (median 66 years [range: 27-91 years], median body mass index 27.3 kg/m^2 ^[range: 13.9-46.9 kg/m^2^]) and 5 infants and children aged 1, 8 and 10 months, 2 years and 13 years, respectively, by emergency physicians (n = 72 applications) and paramedics (n = 5 applications) (Figure 1).

The indications for obtaining vascular access are shown in Table 1. In all patients, the consequence of vascular access was the use of either analgetics or narcotics, cardiovascular active drugs (e.g., inotropes, beta blockers, vasoconstrictors, etc.), naloxone, and fluids.

**Table 1:** Indications for EZ-IO^® ^use in 74 patients

Diagnosis	n and (%)
Cardiac arrest	41 (56%)

Multiple trauma	15 (20%)

Myocardial ischemia	5 (7%)

Pulmonary edema	4 (5%)

Drug poisoning	4 (5%)

Stroke/Intracerebral hemorrhage	3 (4%)

Gastrointestinal hemorrhage	2 (3%)

Needle placement was successful at the first in all except 2 adults. One had undergone total knee arthroplasty which the emergency physician in charge was unaware of, and multiple attempts to place the intraosseous needle into the proximal tibia failed. In the second patient needle insertion was successful *per se*, but the needle was obstructed by osseous chippings, and another IO needle was placed into the other tibia. No immediate procedure-related complications were observed.

The EZ-IO^® ^was used as first-line vascular access device in ten patients (indications: anticipated difficult venous access due to a history of intravenous drug abuse, n = 4; vascular collapse due to hypovolemic shock, n = 3; and cardiac arrest, n = 3). In the remaining 64 patients, a median of three (range 1-12) attempts of peripheral venous cannulation failed before IO access was established.

22 patients were conscious and alert during needle placement. Although the insertion site was not anesthetized and none of the patients described the needle insertion as painful, 18 of them reported pain upon fluid administration via the needle, among them one patient with multiple injuries in whom fluid resuscitation needed to be stopped because of massive intramedullary pain, and a central venous catheter was inserted. Lidocaine 20-40 mg was given to all 4 patients who did not complain about injection pain, and to eight of 18 patients who complained.

The rescuers' subjective rating regarding handling of the device and ease of needle insertion, as described by an analogue scale (0 = entirely unsatisfied, 10 = most satisfied), provided a median score of 10 (range 1-10). The previous personal level of experience with IO needle placement is shown in Table 2.

**Table 2:** Previous personal experience with IO needle placement

Previously placed intraosseous needles (n = 63 EP/PM)*			
**None**	**1-5**	**6-10**	**> 10**

25 (40%)	32 (50%)	3 (5%)	3 (5%)

Data are number and (%)

EM: emergency physician PM: paramedic

* Regardless of device

## Discussion

The EZ-IO^® ^proved as a feasible, effective and readily available vascular access device in the prehospital setting and in the hands of novice users, regardless if emergency physicians or paramedics. IO access was established on the first attempt in 97% and failed only once in a patient with total knee arthroplasty, which can be attributed to the lack of experience with intraosseous needle placement. The emergency physician in charge did not remember one key message of the theoretical and hands-on training: choosing a different access site when needle placement has failed at one site. In another patient the needle was apparently placed correctly, but got obstructed by bony debris. Subsequent needle placement into the other tibia was uneventful.

Our results are in accordance with another recent report on the EZ-IO^® ^device [3]. We estimate that in half of our missions vascular access was required, resulting in a rate of EZ-IO^® ^use of 0.40% (one per 250 patients requiring vascular access). In a recent study on French mobile intensive care units Gazin et al. reported on 4,666 patients who required prehospital vascular access, among them 30 patients in whom the EZ-IO^® ^was used (0.64%; one per 156 patients). The success rate was 84% (first attempt) and 97% (maximum of two attempts) [3]. Both investigations were performed in an emergency physician-dominated rescue system using the same novel device after a brief training. Because difficult vascular access was a relative rarity in both studies, the total number of IO needle placements is low compared to the total number of patients who required vascular access. Performing a single study that includes a representative number of patients in whom out-of-hospital IO access was attempted would probably require hundreds of thousands of patients. Therefore, pooling data from several smaller studies conducted in comparable settings seems to be the most practical approach to reach a sound conclusion regarding safety and efficacy of a new, but rare procedure.

Several techniques can be used to insert IO needles, the best established being manual needles and the spring-loaded driven BIG. The semi-automatic EZ-IO^® ^is another access device which is becoming increasingly popular. Studies in animal and human cadavers demonstrated the superiority of the EZ-IO^® ^over both, the manual needle and the BIG, regarding successful insertion on the first attempt (manual needle 79.5% versus EZ-IO^® ^97.8%; BIG 69.0% versus EZ-IO^® ^96.6%) [10,11]. When the EZ-IO^® ^was compared to the BIG in 40 patients requiring in-hospital cardiopulmonary resuscitation, IO access was established more often (90% versus 80%) and faster (1.8 ± 0.9 min versus 2.2 ± 1.0 min) with the EZ-IO^®^, although these differences did not reach statistical significance [7]. Sunde et al. also found that the EZ-IO^® ^- when used by emergency physicians - had a higher overall success rate than both, the BIG and the manual needle, and an even significantly higher success rate on first attempt [4].

Pain upon infusion and drug administration was observed in 18 of 22 responsive patients, regardless of the fact that 8 of them received 20-40 mg lidocaine via the needle before the infusion was started. However, the 4 patients who did not complain about pain upon injection had all been given IO lidocaine immediately after needle placement. In a study by Cooper et al., 32 needles were inserted in combat casualities in Afghanistan, always using the EZ-IO^®^. Pain was observed in all responsive patients with the pain of infusion exceeding that of the underlying injuries in 3 cases [5]. We also observed one patient with multiple injuries in whom the infusion needed to be stopped because of unbearable pain. The manufacturer recommends lidocaine administration, 20-40 mg in adults and 0.5 mg/kg in children, to prevent such pain [13]. If this is the optimal dose, however, needs to be further studied. Nevertheless, based on the experiences made in our study all emergency physicians and paramedics are now explicitly advised to first administer lidocaine in all conscious patients before injecting any other drug or administer fluids.

There is an ongoing debate if vascular access should be established at all costs in the prehospital setting. Multiple attempts to achieve IV access may prolong the prehospital treatment time, which can be crucial especially in trauma patients. We therefore recommend that IO access should be immediately considered whenever a.) vascular access has an impact on the prehospital treatment regimen of the patient and b.) peripheral venous access requires more than two attemps or takes longer than 90 seconds in such patients.

### Study limitations

The study has several limitations. It is an observational cohort study, but did not compare different devices for IO access in randomized fashion. Furthermore, the decision for IO access was solely made at the discretion of the rescuer in charge. No conclusion can therefore be drawn regarding the efficacy and the potential benefits of a prehospital "difficult vascular access algorithm" consisting of both, IV access and alternative measures. Finally, we did not assess the pain scores during intraosseous infusion/injection in conscious patients. Flow rates of fluids were also not measured, and no late complications (e.g., osteomyelitis, inflammation, etc.) were assessed.

## Conclusion

We demonstrated that the EZ-IO^® ^intraosseous needle driver was an efficient alternative to establish immediate out-of-hospital vascular access. However, pain upon intramedullary infusion was observed in the majority of responsive patients. If lidocaine administration, as suggested by the manufacturer, significantly reduces injection pain needs to be further studied.

## Competing interests

None of the authors has any conflict of interest with products and/or companies mentioned in the manuscript that could potentially bias their work. There were no external sources of funding.

## Authors' contributions

RS, LL, and CB conceived and designed the study. GL, KZ, and CB collaborated on the article and were responsible for statistical analysis. GL collaborated on background research. RS, US, and LL collected data. CB takes responsibility for the paper as a whole. All authors have read approved the final manuscript.
